# CALENDAR

**DOI:** 10.1038/sj.bjc.6690504

**Published:** 1999-04


					1113 April 1999
MICRO 2000 International Microscopy Exhibition and
Conference
London, UK
Further information from:
Alison Winton, Exhibition Organizer, Royal Microscopical
Society, 37/38 St Clements, Oxford OX4 1AJ, UK.
E-mail: exhibitions@rms.org.uk
16 April 1999
Joint Meeting of the Section of Urology, Royal Society
of Medicine and the British Prostate Group - Prostate
Cancer: The Impact of Basic Science on Screening,
Treatment and Quality of Life
London, UK
Further information from:
Meeting Secretariat, c/o British Association of Urological
Surgeons, 35/43 Lincoln's Inn Fields, London WC2A 3PN, UK.
Tel: +44 (0) 171 405 1390, Fax: +44 (0) 171 404 5048
1719 May 1999
Radiology 1999-Imaging, Science & Oncology
International Convention Centre, Birmingham, UK
Proferred papers are invited for poster presentation. Deadline for
Work in Progress: 22 February 1999
Further information from:
Radiology 1999 Secretariat, 36 Portland Place, London W1N 4AT,
UK. Tel: +44 (0) 171 307 1410, Fax: +44 (0) 171 307 1414
Erratum
Br J Cancer 79(7/8): 1288-1301
Cancer mortality and morbidity among plutonium
workers at the Sellafield plant of British Nuclear Fuels
RZ Omar, JA Barber and PG Smith
The correct version of Table 1 appears below:
CALENDAR
1946
British Journal of Cancer (1999) 79(11/12), 1946
(c) 1999 Cancer Research Campaign
Article no. bjoc.1998.0312
Table 1 Study population at 1 January 1993
Men (%) Women (%) Total (%)
Total number of workers 11 679 (100.0) 2 689 (100.0) 14 385 (100.0)
No. for whom date of birth
not known 44 (0.4) 5 (0.2) 66* (0.5)
No. eligible for analysis 11 635 (99.6) 2 684 (99.8) 14 319 (99.5)
Alive 1 January 1993 7 809 (66.9) 2 139 (79.5) 9 948 (69.2)
Died before 1993 3 410 (29.2) 44 (16.5) 3 854 (26.8)
Emigrated 381 (3.2) 85 (3.2) 466 (3.2)
Untraced 35 (0.3) 16 (0.6) 51 (0.3)
Total years of follow-up 332 532.4 82 899.3 415 431.6
Average duration of
follow-up (years) 28.6 30.9 29.0
*Includes 16 of unknown sex.

				

